# 316 Culture Change: Implementing and Sustaining Best Practices for Blood Culture Collection

**DOI:** 10.1017/ash.2026.10453

**Published:** 2026-06-23

**Authors:** Hye-Jin Ki, Ji Young Lee, Bomun Lee, Hyukmin Lee, Ji-Man Kang, Soo Yeon Kim, Young-Ju Han, Hamin Kim, Seok-Jin Lee

**Affiliations:** 1 Severance Hospital, Yonsei University College of Medicine; 2 Yonsei University College of Medicine; 3 severance hospital; 4 Severance Hospital, Yonsei University; 5 Department of Pediatrics, Severance Children’s Hospital, Yonsei University College of Medicine; 6 Severance Hospital

## Abstract

**Background:** The World Health Organization (WHO) designates Candida auris as a critical priority fungal pathogen due to its high multidrug resistance, transmissibility, and invasiveness, particularly in critically ill hosts, including the pediatric population. Here, we report the successful termination of a prolonged C. auris outbreak in a Pediatric Intensive Care Unit (PICU) using a multifaceted intervention strategy, including whole-genome sequencing (WGS) and unit-level clinical density reduction. **Methods:** This study was conducted in a 10-bed PICU at a 2,172-bed tertiary hospital in South Korea. Following an incidence surge in the second quarter of 2025, we implemented an intensive bundle in June 2025: (1) Surveillance: Point Prevalence Surveys (PPS), environmental cultures, and WGS to identify transmission dynamics; (2) Environmental Control: Terminal cleaning with 5,000 ppm sodium hypochlorite, daily cleaning frequency increased to three times daily with additional staffing, and real-time healthcare personnel (HCP) adherence monitoring; (3) Source Control: Active relocation of colonized patients to general wards to reduce PICU clinical density. **Results:** From January 2023 to June 2025, 28 C. auris isolates were identified from 27 patients. Urine was the primary source (n=20, 71.4%; 85.0% with indwelling urinary catheters), and four cases (14.3%) were candidemia. Incidence density peaked in June 2025 (13.89 per 1,000 patient-days) following a PPS that revealed a 28.6% positivity rate. Environmental cultures (monitor cables, infusion pumps) remained positive despite rigorous disinfection. WGS analysis identified all isolates as belonging to Clade I, consisting of two clusters with 100% resistance to fluconazole and amphotericin B. Genomic analysis further revealed that Cluster 2 was restricted to the PICU, confirming unit-specific reservoirs. To mitigate density, relocation was pursued for eight cases; five (62.5%) were transferred within five days of result reporting (range: -1 to 5 days). Despite delayed relocation in three cases (up to 76 days) due to clinical instability, no secondary transmissions occurred in general wards. Following these interventions, environmental cultures became negative, and the incidence rate dropped to zero through October 2025. **Conclusions:** C. auris can survive for prolonged periods on medical devices despite intensified protocols, including repeated 5,000 ppm terminal disinfection. Given this persistence, a multidisciplinary approach, coordination, and sustained adherence among HCP in the ICU are essential for successful outbreak control. Furthermore, active patient redistribution served as an effective unit-level source control strategy by reducing clinical density. The integration of genomic data was crucial for identifying unit-specific transmission patterns and validating the unit-targeted protocols.

